# Editorial: Comprehensive approaches to multivalvular disease: diagnosis, treatment, and innovation

**DOI:** 10.3389/fcvm.2026.2001254

**Published:** 2026-09-30

**Authors:** Alon Shechter, Anastasios Apostolos

**Affiliations:** 1 Department of Cardiology, Rabin Medical Center, Petach Tikva, Israel; 2 Gray Faculty of Medical & Health Sciences, Tel Aviv University, Tel Aviv, Israel; 3 Department of Cardiology, Royal Brompton Hospital, Royal Brompton and Harefield Hospitals, Guy’s and St Thomas’ NHS Foundation Trust, London, United Kingdom; 4 Faculty of Medicine, Imperial College London, London, United Kingdom

**Keywords:** diagnosis, echocardiagraphy, innovation, risk stratificacion, valvular heart disease

Valvular heart disease remains a major challenge in contemporary cardiology, owing in part to the complex interplay among loading conditions, filling pressures, cardiac remodeling, and ventricular function, which contributes to substantial variability in clinical presentation and disease progression. Further, the assessment and management of multivalvular disorders is substantially less well defined than those of isolated valvular lesions, potentially leading to mis-treatment. Our current Research Topic aims to address these challenges by bringing together contemporary work spanning novel diagnostic technologies, perioperative risk stratification, and advanced echocardiographic phenotyping. The four manuscripts published within this Research Topic illustrate innovative directions in the evolving field of valvular heart disease, while highlighting areas in which additional research is needed.

The first article, by Li et al., deals with intraoperative hemodynamic assessment during valve replacement surgery. In a retrospective matched cohort of 162 patients, the investigators compared transesophageal echocardiography (TEE) combined with FloTrac monitoring with TEE accompanied by conventional invasive arterial pressure monitoring. Significant differences between monitoring strategies were observed in several hemodynamic variables, including cardiac output, cardiac index, stroke volume variation, and their temporal behavior during surgery. However, despite the greater hemodynamic detail provided by TEE plus FloTrac, postoperative complication rates, including cognitive decline, did not differ significantly between groups, suggesting that an intraprocedural physiological measurement does not necessarily translate into improved clinical outcomes. Further prospective studies are needed to investigate the prognostic impact of monitoring protocols during valvular interventions.

The second paper, by Close et al., reports on the detection of isolated aortic stenosis using auscultation recordings obtained with unmodified mobile phones and analyzed by computational nonlinear dynamics. In this single-center pilot study of 248 participants, the best-performing model for aortic stenosis achieved an area under the curve of 0.872 and a sensitivity of 92.3%. Interestingly, recordings obtained by study personnel and by participants themselves appeared to be equally informative, suggesting feasibility of patient-generated recordings. The implications of this work extend beyond aortic stenosis itself, as access to definitive diagnostic tests requiring appropriately trained personnel is limited in general, and the need for low-cost screening tools is as high as ever. While thought-provoking, the study's findings require external prospective validation across broader populations as well as evaluation of whether such screening improves referral, diagnosis, treatment, and outcomes.

The third article, by Gharehdaghi et al., focuses on risk stratification in isolated tricuspid regurgitation (TR). In this multicenter retrospective study of of 104 patients with at least moderate TR, the TRI-SCORE independently forecasted the risk for all-cause mortality and/or heart failure hospitalizations following both conservative and invasive (i.e., surgery or transcatheter edge-to-edge repair) management. Moreover, one-year mortality increased progressively across low-, intermediate-, and high-risk categories, from 7% to 13% and 33%, respectively. These findings are particularly relevant, as proper risk stratification is essential for successful treatment and the avoidance of a futile one. Yet, the absence of a significant survival difference between treatment groups in this observational cohort highlights the limitations of non-randomized comparisons.

Last but not least, Liao et al. explore advanced echocardiographic markers in medically managed secondary mitral regurgitation. Among 399 patients followed for a median of 35 months, both impaired left atrial reservoir strain and global work efficiency (GWE) correlated with a higher risk for all-cause mortality. Using mediation analysis, the authors concluded that GWE accounted for approximately 16% of the association between left atrial reservoir strain and mortality. This study exemplifies the ongoing transition from simply measuring the valve lesion itself to characterizing its “downstream” consequences on the myocardium, especially when conventional measures (e.g., left ventricular ejection fraction) might not fully capture overt ventricular/atrial dysfunction. The retrospective, single-center design and observational nature of the analysis warrant caution regarding causal interpretation, calling for prospective validation.

Taken together, the four articles assembled in this Research Topic demonstrate the breadth of innovation occurring in contemporary valvular cardiovascular medicine. Multimodality intraoperative monitoring aims to improve physiological characterization; digital auscultation seeks to facilitate earlier detection; risk scores are used to refine treatment selection; and advanced echocardiographic indices are used to uncover clinically meaningful cardiac dysfunction beyond conventional measurements. The studies emphasize a continued greater objective, which is to identify the right patient at the right stage of disease and to determine the intervention most likely to alter his/her trajectory.

